# Telemedicine or In-Person: Referral Letter Content Influencing How a Patient Receives Treatment

**DOI:** 10.1089/tmr.2022.0006

**Published:** 2022-05-03

**Authors:** Edwin Phillip Greenup, Matthew Page, Daniel Best, Stephanie Ferdinands, Natalie Atkins

**Affiliations:** ^1^ Clinical Excellence Queensland, Queensland Health, Brisbane, Australia.; ^2^ Darling Downs Hospital and Health Service, Toowoomba, Australia.

**Keywords:** referral, outpatient, referral letter, clinician acceptance, clinician attitudes, delivery modality

## Abstract

**Objective:**

This study investigated hospital-based specialist services that provide both traditional hospital outpatient appointments (in-person) or through a live videoconferencing session (telehealth) to referred patients. Referral letters submitted to these clinics were assessed against an inclusion criterion and grouped according to which of delivery method the patient received for their appointment (in-person or telehealth). These groups were then compared for differences to see what factors, if any, influence the likelihood of a patient being offered a telehealth appointment.

**Methods:**

An extract of all referral letters meeting inclusion criteria between July 01, 2019 and June 30, 2020 were collected (*n* = 441). Letters were grouped according to delivery modality (in-person or telehealth) and differences between the groups, including variables such as patient demographics, clinical condition, and urgency and the reviewing clinician were assessed for associations.

**Results:**

This study observed that where the referring clinician suggested a telehealth appointment for their patient, this was more likely to be offered (38.25%) compared with referrals that did not (7.36%) (*x*^2^_1_ _=_ 28.33, *p* = 0.1857, odds ratio = 2.77). Patients were more likely to be offered a telehealth appointment the further they lived from the treating facility (*T =* −4.51 on 106.59 df, *p* = 1.622 e-05). Variation in the selection of delivery modality among reviewing clinicians was also observed (*x*^2^_1_ = 42.334, *p* < 1.42e-08).

**Discussion:**

Existing research indicates there is a strong link between the perceptions clinicians as individuals have of telehealth and a willingness to offer this modality to patients. Despite this, specific information about a patient contained within a referral letter may influence the delivery modality that the patient will be offered for their initial appointment. It is important that this information is more routinely included in letters sent by referring clinicians to hospital-based specialist services. It is equally important that when included, this information is identified and actioned by reviewing clinicians in a consistent way. Doing so will benefit patients by increasing the likelihood that they will receive specialist outpatient care in a manner that suits them best.

## Introduction

**FIG. 1.**
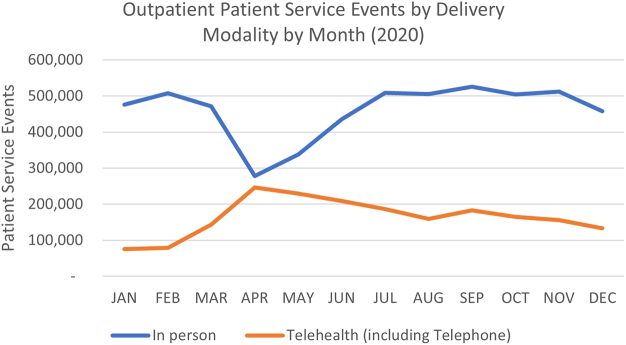
Outpatient service events by modality, by month, whole of organization (Queensland Health), 2020 calendar year, capturing the peak of the telehealth usage in response to the COVID-19 pandemic. COVID-19, coronavirus disease 2019.

The reliance on telehealth to deliver outpatient appointments at the beginning of the coronavirus disease 2019 (COVID-19) pandemic demonstrates that these models of care have reached a maturity and capacity to serve more patients than they ever had before.^1,2^ The subsequent plateau and retreat from the peak levels of activity (Fig. 1), despite broad support by health consumers,^3–6^ indicate that telehealth has not become a genuine alternative to in-person appointments for many public hospital departments across Queensland, Australia.

With sustained dedication from clinicians required to transition from exclusively offering traditional hospital outpatient appointments (in-person) to also include telehealth, embedding these services is likely to remain a persistent challenge regardless of periods of peak demand such as those experienced recently.

This study sought to investigate specialist clinics that reported providing telehealth services to patients but in a nonsystematic or *ad hoc* way (i.e., telehealth was offered based on clinician judgment alone and not mandated by hospital administration). In these settings, it was speculated that the decision to offer an in-person or telehealth appointment was made based on the content of the referral letter sent to the clinical department, reviewed by a clinician before scheduling an initial appointment.

Focusing on the referral letter as the first contact a public specialist service will have with a patient, this study seeks to understand why some patients are offered telehealth appointments, whereas others are not, even within the same hospital or clinic. The researchers involved in this exercise found no existing analysis of referral documentation to determine the mode of appointment a patient is likely to receive.

## Methods

A single public hospital in inner regional Queensland with a history of providing both in-person and live, two-way videoconference (telehealth) outpatient appointments for >20 years to patients living in urban and rural locations throughout their traditional geographic catchment was selected to conduct this study. A record of referral letters that were received by the central referral hub between July 01, 2019 and June 30, 2020 was compiled. This list was assessed against the following inclusion criteria:

The patients
(1) had received their initial appointment by December 31, 2020;
(2) were referred to a specialist service that does not have any existing referral criteria or guidelines for reviewing clinicians to follow;
(3) were referred to a specialist service that delivered at least 20% of their total outpatient activity through telehealth models of care;
(4) were referred to a specialist service that employed at least two different medical officers that reviewed referral letters and determined that they would see the patient in either a telehealth or in-person appointment; and
(5) were new to the public health service (i.e., they did not have any existing medical information stored in their medical record that a reviewing specialist could access).

These criteria were designed to produce a cohort of referrals in which the patient has a chance of being offered either an in-person or telehealth appointment and the content of the referral letter as well as the individual preferences of the reviewing clinician would determine the delivery modality a patient would receive. Four hundred forty-one from a total of 2178 referral letters met the aforementioned criteria of which 338 patients received an in-person appointment and 103 a telehealth appointment from pediatrics, gastroenterology, and endocrinology specialties. Referrals were submitted by 117 different referring clinicians from 108 referring sites.

In addition to the delivery modality a patient received, a set of variables were gathered from these referral letters, including the patient's age, address (postcode only), distance to hospital (km), triage category, primary diagnosis made by referring clinician, whether the referral was from another clinic internal or external to the hospital (i.e., general practitioner), whether the patient's preference for telehealth was mentioned in the referral, and whether the referral was named (i.e., addressed to a specific doctor as opposed to a clinic). The clinician who reviewed their referral was also recorded. The entire content of the letter was also scanned with optical character recognition (OCR) software (Adobe Acrobat Professional 2017) and the digitized output passed through Text Pattern Recognition Software (Leximancer LexiPortal V5 Academic). Patient or clinician-identifiable information was replaced with a code known only to researchers and the retrospective nature of this medical record review.

## Results

**FIG. 2.**
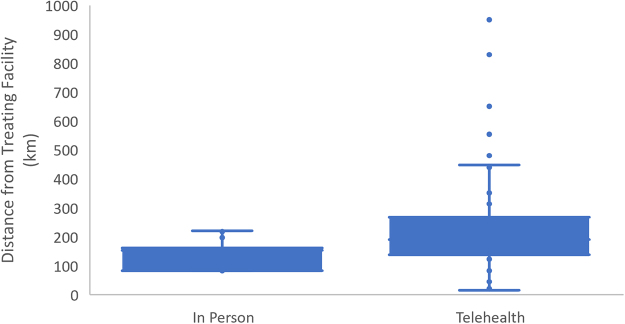
Mean distance between treating facility and patient address, by modality.

A null hypothesis that the patient's distance from hospital would be the same for in-person and telehealth groups was established and tested using Welch's *t*-test. The results of this test indicate the average distance from the hospital for in-person patients was closer than the average distance for telehealth patients (Fig. 2). (*T* = −4.51 on 106.59 df, *p* = 1.622 e-05).

To establish whether an association exists between the categories of delivery modality and the medical officer reviewing the referral, doctors who had reviewed less than five referrals were removed and a chi-square test of independence was performed. This test established an association between delivery modality and reviewing clinician (*x*^2^_1_ = 42.334, *p* < 1.42e-08).

A similar chi-square test of independence determined that where the referring clinician suggested a telehealth appointment for their patient, it was more likely to be received (38.25%) compared with referrals that did not (7.36%) (*x*^2^_1__=_ 28.33, *p* = 0.1857, odds ratio = 2.77).

Further tests found no association between delivery modality and triage category (*x*^2^_2__=_ 5.9916, *p* = 0.05), or if the referral was addressed to an individual doctor as opposed to a department (*x*^2^_1_ = 0.364, *p* = 0.546).

Analysis of the digitized text of the referrals using the pattern recognition software identified several related word terms (concepts), which appear in varying frequencies among both the referrals when grouped by modality. Several of these appear more frequently in referrals that resulted in in-person appointments, including “opinion” (*n* = 136, 40.2% of in-person referrals vs. *n* = 28, 27.18% of telehealth referrals) and “diagnose” (*n* = 35, 10.4% of in-person referrals vs. *n* = 7, 6.8% of telehealth referrals).

Conversely, concepts including “history” (*n* = 102, 30.18% of in-person referrals vs. *n* = 45, 43.69% of telehealth referrals) and “review” (*n* = 106, 31.36% of in-person referrals vs. *n* = 56, 54.39% of telehealth referrals) appear more frequently in referrals that resulted in telehealth appointments. Referral letters ranged in length from 32 to 5955 characters (mean = 750 characters).

## Discussion

Telehealth proof of concepts are often initiated by an individual or small group of clinicians that are passionate about the potential patient benefits they offer. To transition from this state to an established service where a clinical department provides both in-person and telehealth delivery modalities requires consideration of some administrative processes, including how referrals will be assessed and appointments scheduled. Several strategies have been proposed in response to this challenge. These include a “telehealth first” or “digital by default” approach where referrals will be assumed to be suitable for a telehealth appointment and in-person appointments are offered by exception where clinicians reviewing the referral would prefer to assess the patient in-person.

This approach is effective for integrating telehealth into clinical practice and has the added advantage of more equally offering telehealth appointments to metropolitan and rural patients. As a policy, however, offering telehealth as the default delivery modality can be a radical change to a clinic familiar with delivering traditional in-person outpatient appointments. Particularly during times when a clinical department is facing pressure to reduce cost and drive down waiting lists, implementing this policy to achieve less tangible outcomes such as improved patient satisfaction or improving equity of access to services requires enormous commitment.

Telehealth provider directories are sometimes employed to promote specialist services to referring clinicians, or directly to patients, that can deliver telehealth appointments.^7,8^ Telehealth provider directories can be easy to implement although may have a higher burden to maintain depending on the level of detail they contain. A high degree of granularity may be required for a telehealth provider directory to be a useful tool for referring clinicians.

It could be necessary to include not only the treating facility name and clinic type, but also the names of all staff and visiting specialists, subspecialties, referral criteria, and so on. Even if this document is well maintained, the decision of whether a patient is appropriate for a telehealth appointment remains that of the clinician reviewing a received referral not what is stated in a provider directory.

A potential alternative that addresses the potential downsides to both described strategies is implementing a manual or automatic review process of all referrals before their assessment by a receiving clinician. This approach attempts to assist, rather than remove, clinical input in decision making by flagging any content that may indicate a referred patient is particularly suited to a telehealth appointment (i.e., location of residence or personal preference).

This study has demonstrated that these details are relevant to reviewing clinicians when they are determining how a patient will receive their appointment. A referral containing content of interest that has been flagged before being assessed by a reviewing clinician is likely to aid reviewing clinicians and by treating these as in-patient by exception, would also increase the likelihood that referring clinicians, on behalf of their patients, receive the type of appointment that best meets their needs.

**FIG. 3.**
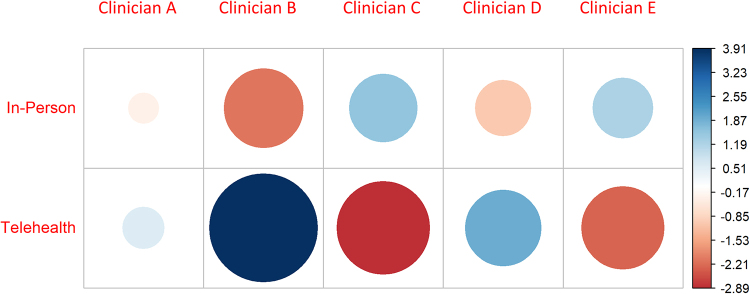
Strength (large = strong, small = weak) and direction (*blue* = more likely to offer, *red* = less likely to offer) the correlation coefficient between the five reviewing clinicians involved in this study and the modality of appointment a patient received.

**FIG. 4.**
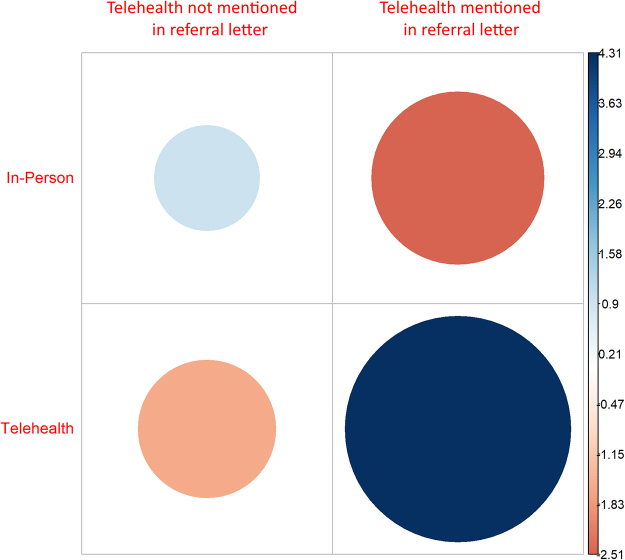
Strength (large = strong, small = weak) and direction (*blue* = more likely to receive, *red* = less likely to receive) of the correlation coefficient between delivery modality of the appointment a patient received based on whether telehealth was mentioned in the referral letter.

Existing research indicates there is a strong link between the perceptions clinicians as individuals have of telehealth and a willingness to offer this modality to patients.^9–11^ This observation was supported by the variation in likelihood a clinician reviewing referral letters would assign a patient an in-person or telehealth appointment (Fig. 3). Importantly, this study observes that although these perceptions exist, they can be influenced by prompting clinicians reviewing a new referral that telehealth delivery modes exist and may be appropriate for this patient (Fig. 4).

Researchers were unable to determine whether there are differences in overall referral letter content through text pattern analysis of the referral letters. Similar concepts emerge in both letters that resulted in an in-person or telehealth initial appointment. More frequently observed concepts in in-person referrals (opinion, diagnose) compared with more frequently observed concepts in telehealth referrals (history, review) may indicate that telehealth was used more regularly for the management or second opinion of an existing, chronic condition, although in a patient not previously seen by the clinic. In-person appointments may be preferred by clinicians where patients are presenting with undiagnosed conditions. This topic would benefit from a larger scale more targeted study.

### Limitations

Analysis was performed on referral letters gathered from a limited number of specialist services at one hospital within a large public health service. The results described earlier are applicable to this data set and the authors make no claim to their generalizability. The authors of this study acknowledge it was not possible to determine whether variation in telehealth appointment selection among reviewing clinicians was due to an inconsistent assessment of clinical risk or personal preference for one delivery modality over another. Patient demographic information including age and gender were not used in the analysis nor were potentially relevant details of the medical officers involved, including age, gender, and years in practice.

## Conclusion

Referral letters to hospital-based specialist services vary in length and content. This study demonstrates that key pieces of information, including content that is not clinical in nature, are often identified by reviewing clinicians. This information, including the patient's location and their preference (or referring clinicians' recommendation) for how they receive their appointment, can influence the type of appointment a patient receives. Clinicians were more likely to offer a telehealth appointment to patients who live at greater distances from hospital or where the possibility of a telehealth appointment was mentioned in the referral letter, regardless of their perceptions of delivering care by this modality.

Incorporating a telehealth delivery modality to a specialist outpatient department adds complexity to what is already a complex process. Encouraging referring clinicians to advocate for a preferred delivery modality on behalf of their patient and applying techniques to ensure these requests are consistently identified by clinicians reviewing referrals, are processes that are likely to benefit both patients and clinicians involved in the referral process.

## Abbreviations Used

COVID-19: coronavirus disease 2019
HREC: Human Research Ethics Committee
OCR: optical character recognition
